# Supplementary material for: Intrinsic negative magnetoresistance from the chiral anomaly of multifold fermions
Source: Nat Commun. 2024 Aug 2;15:6526. doi: 10.1038/s41467-024-50451-5 (PMC11297145; doi:10.1038/s41467-024-50451-5)
Supplement: Supplementary file 1 — Supplementary Information [file 41467_2024_50451_MOESM1_ESM.pdf]

Supplementary Information for
Intrinsic negative magnetoresistance from the chiral anomaly of multifold fermions

F. Balduini,[1] A. Molinari,[1] L. Rocchino,[1] V. Hasse,[2] C. Felser,[2] M. Sousa,[1] C. Zota,[1] H. Schmid,[1] A. G. Grushin,[3] and B. Gotsmann[1]

[1]*IBM Research - Zurich, 8803 Ruschlikon, Switzerland*

[2]*Max Planck Institute for Chemical Physics of Solids, 01187 Dresden, Germany*

[3]*Univ. Grenoble Alpes, CNRS, Grenoble INP, Institut Néel, 38000 Grenoble, France*

I. SUPPLEMENTARY NOTE 1

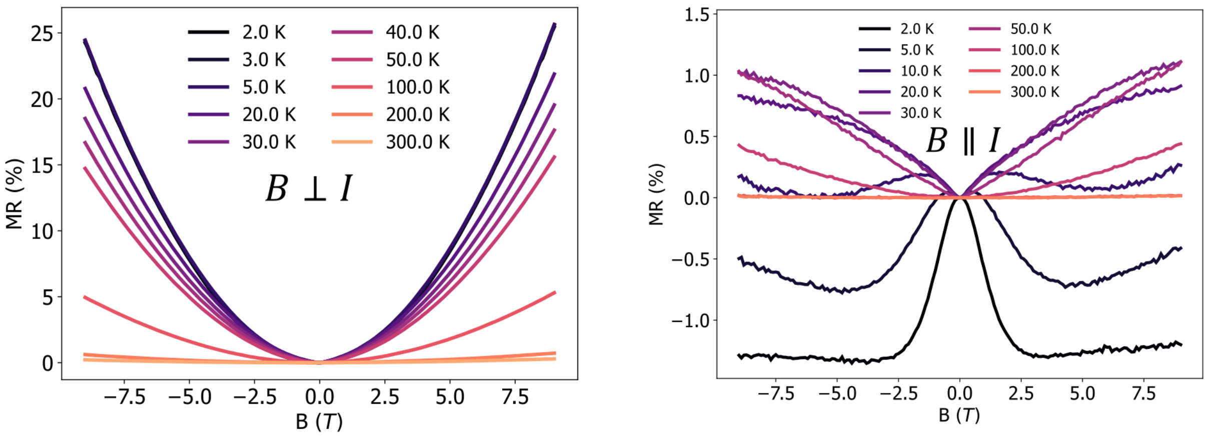

Supplementary Figure 1: **Temperature dependence of the MR.** Transverse MR $B \perp I$ (**left**) and longitudinal MR $B||I$ (**right**).

II. SUPPLEMENTARY NOTE 2

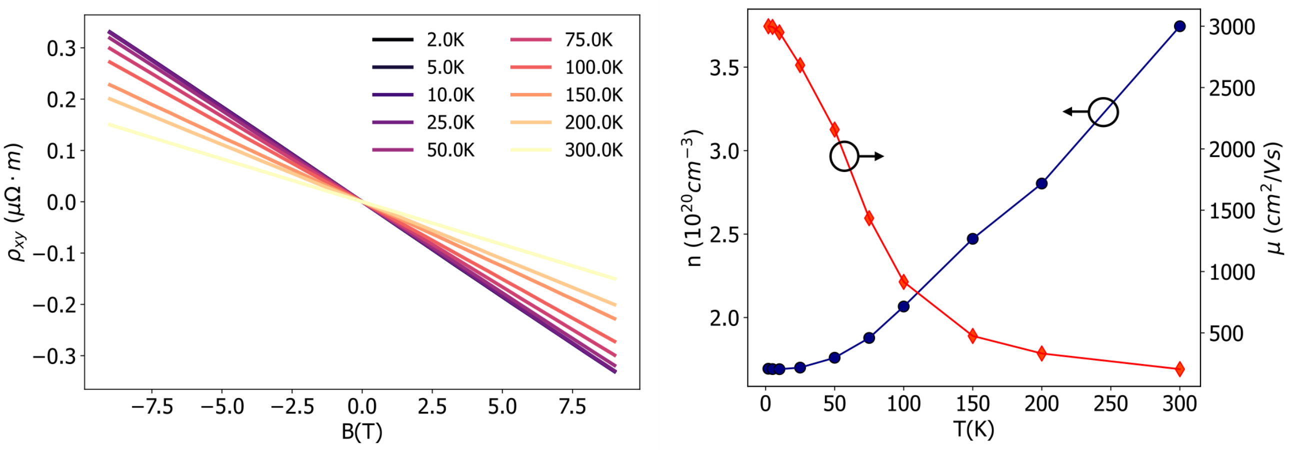

Supplementary Figure 2: **Hall effect** Hall resistance (**left**) and carrier density and mobility extracted from a linear fit of the Hall effect $\rho_{xy} = \frac{B}{ne}$, where n is the carrier density and e the electron charge. The mobility is calculated as $\mu = (ne\rho_{xx})^{-1}$ (**right**).

III. SUPPLEMENTARY NOTE 3

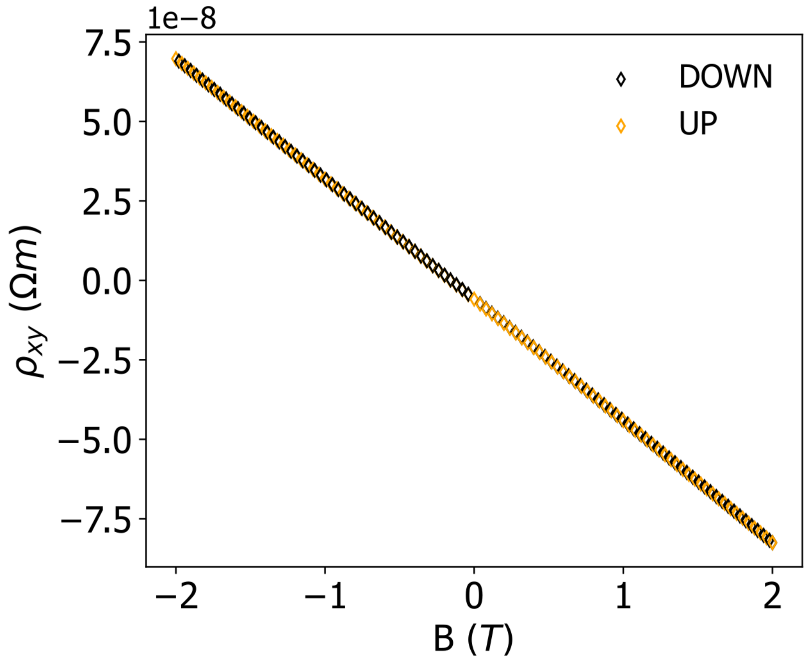

Supplementary Figure 3: **Bidirectional Hall effect measurement.** The Hall signal does not show any hysteresis, supporting the hypothesis of negligible magnetic impurities in the sample.

IV. INTRODUCTION TO MULTIFOLD FERMIONS IN COSI

Higher-spin fermions have total monopole charges at the Fermi level of $1 < |C| \leq 4$ [1, 2], generally surpassing Weyl nodes, which have $|C| = 1$. As we review next, the chiral-anomaly-induced negative magnetoresistance is proporional to C, and thus it is larger for higher-spin fermions than for a single Weyl nodes, given the same Fermi velocity.

The Hamiltonian of a generic chiral multifold fermion of (pseudo)spin S around a time-reversal invariant momentum, is, to lowest order in momentum $\mathbf{k}$, given by

$$H_{\mathbf{k}} = v\mathbf{k} \cdot \mathbf{S}(\alpha). \tag{S1}$$

The vector $\mathbf{S}$ is composed out of of three matrices that depend in general on band-structure parameters, α, and do not form a spin-S representation of SU(2). However, neglecting spin-orbit coupling, as pertinent for CoSi, forces the matrices in $\mathbf{S}(\alpha)$ to realize a representation of the spin algebra of spin-s. Which may choose our convention such that, to linear order in momentum, the band structure at the Γ point is given by $\epsilon_S = m_S v|\mathbf{k}|$ and $\epsilon_S = 2m_S v|\mathbf{k}|$ for integer and half-integer spin, respectively, with $m_s = -S, -S+1, \cdots, S-1, S$, and v the Fermi velocity.

At the Γ point and without spin-orbit coupling the three-fold irreducible representation (irrep) of the little group of CoSi at the Γ point enforces that $\mathbf{S}$ is a representation of 3×3 spin-1 matrices[1, 2]. Hence, to linear order in momentum, the band structure at the Γ point is given by $\epsilon_\Gamma = m_1 v_\Gamma |\mathbf{k}|$ with $m_1 = -1, 0, +1$. The Berry curvature of band m_1 is given by $\mathbf{\Omega}_{\mathbf{k}}^{m_1} = m_1 \frac{\mathbf{k}}{|\mathbf{k}|^3}$, corresponding to a charge $C = \pm 2$ monopole for bands $m_1 = \pm 1$ and charge $C = 0$ monopole for band $m_1 = 0$.

At the corner of the Brillouin zone $R = (\pi, \pi, \pi)$ a four-fold irrep enforces a double spin-1/2 Weyl fermion with Hamiltonian

$$H_{\mathbf{k}} = v_R \mathbf{k} \cdot \sigma \otimes \tau_0, \tag{S2}$$

where τ_0 encodes the Weyl copies at R. The band structure is given by two doubly-degenerate Weyl dispersion relation $\epsilon_R = \pm v_R |\mathbf{k}|$. For each copy (there are four counting spin), the Berry curvature of band $\pm$ is given by $\mathbf{\Omega}_{\mathbf{k}}^{\pm} = \pm \frac{\mathbf{k}}{2|\mathbf{k}|^3}$, corresponding to charge $C = \pm 1$ monopoles.

The chiral anomaly of higher-spin fermions is proportional to its monopole charge C. A monopole charge of C implies that the chemical potential will cross C chiral Landau

levels [1, 3]. Following the arguments by Nielsen and Ninomiya [4, 5], an electric field $\mathbf{E}$ with a component parallel to the direction of magnetic field $\mathbf{B}$ will increase or decrease the chemical potential of positively or negatively dispersing chiral Landau levels, respectively. Both contributions add up and hence, the chiral anomaly equation for a single multifold fermion of charge C becomes

$$\partial_\mu j^\mu = |C| \left(c_A \mathbf{E} \cdot \mathbf{B} \right), \tag{S3}$$

where $j_\mu = (\rho, \mathbf{j})$ is the current four-vector, composed of the charge density ρ and charge current density $\mathbf{j}$, and $c_A = \frac{1}{4\pi^2}$ is the chiral anomaly coefficient in units of $e^2/\hbar$. It has been noted that, when the chemical potential is exactly at the spin-1 crossing point, a linear model predicts that the chiral anomaly is equal to that of a single Weyl node [6]. While this is an interesting regime, it is never achieved experimentally in CoSi. Hence in the following we calculate the chiral-anomaly-induced magneto-conductivity coming from the R and Γ multifold fermions semi-classically.

V. SEMICLASSICAL BOLTZMANN TRANSPORT

This section contains the main theoretical results behind our interpretation of the longitudinal magnetoresistance. The three-fold and the double Weyl fermions, found in CoSi, induce negative magnetoresistance (positive magnetoconductance). As in other topological semimetals, this result is a consequence of their finite Berry curvature. Our calculations include the orbital magnetic moment, which acts to reduce, but not destroy the chiral anomaly induced negative magnetoresistance.

Our calculation generalizes standard derivations of positive magnetoconductance [7–16] for Weyl fermions. The positive magnetoconductance of the double Weyl at the R point follows from these works. The R point is well approximated by two independent Weyl fermions of the same chirality. We repeat this derivation here for pedagogical reasons, including the aforementioned orbital magnetic moment.

As for the Γ point, the positive magneto-conductance can be derived in the ultraquantum, Landau level limit [3]. However, CoSi is far from the ultraquantum regime, and our results need to be understood in the semiclassical regime of low magnetic fields. Ref. [11] derived the positive magneto-conductance of three-fold fermions. However, this reference did not include the orbital magnetic moment. We find that this quantity acts to decrease, but not

overcome, the coefficient of the positive magnetoconductance found in Ref. [11]. The orbital magnetic moment also contributes to a B^3 Hall contribution in the semiclassical regime.

Our derivation below follows closely that of Refs. [12, 16] and uses the same notation. The main differences are that here we will be concerned only with DC response, we will expand the Hall response to cubic order in magnetic field, and we will calculate the responses of threefold fermion and not only a Weyl fermion.

The motion of an electron wavepacket in a metal centred at position $\mathbf{r}$ and momentum $\mathbf{k}$ is governed by the semiclassical equations of motion [17, 18]

$$\dot{\boldsymbol{r}} = \frac{1}{\hbar}\boldsymbol{\nabla}_{\boldsymbol{k}}\epsilon_{\boldsymbol{k}} - \dot{\boldsymbol{k}} \times \boldsymbol{\Omega}_{\mathbf{k}}, \tag{S4a}$$

$$\hbar\dot{\boldsymbol{k}} = -e\boldsymbol{E} - e\dot{\boldsymbol{r}} \times \boldsymbol{B}. \tag{S4b}$$

These equations include the electric and magnetic fields, $\boldsymbol{E}$ and $\boldsymbol{B}$ respectively, the Berry curvature

$$\boldsymbol{\Omega}_{\mathbf{k}} = -\mathrm{Im}[\langle\nabla_{\boldsymbol{k}} u_{\boldsymbol{k}}| \times |\nabla_{\boldsymbol{k}} u_{\boldsymbol{k}}\rangle], \tag{S5}$$

and the orbital magnetic moment

$$\boldsymbol{m}_{\boldsymbol{k}} = -\frac{e}{2\hbar}\mathrm{Im}[\langle\nabla_{\boldsymbol{k}} u_{\boldsymbol{k}}| \times (H_{\boldsymbol{k}} - \epsilon^0_{\boldsymbol{k}})\,|\nabla_{\boldsymbol{k}} u_{\boldsymbol{k}}\rangle]. \tag{S6}$$

with the convention for the electron charge $e > 0$, and $\langle u_{\boldsymbol{k}}|$ is Bloch's wavefunction of a single band. When $\boldsymbol{B} \neq 0$, the magnetic orbital moment shifts the energy dispersion in the absence of magnetic field, $\epsilon^0_{\boldsymbol{k}}$, to

$$\epsilon_{\boldsymbol{k}} = \epsilon^0_{\boldsymbol{k}} - \boldsymbol{m}_{\boldsymbol{k}} \cdot \boldsymbol{B} \tag{S7}$$

where $H_{\boldsymbol{k}}\,|u_{\boldsymbol{k}}\rangle = \epsilon^0_{\boldsymbol{k}}\,|u_{\boldsymbol{k}}\rangle$

The current density $\boldsymbol{j}$ is given by

$$\boldsymbol{j} = -e\int[d\boldsymbol{k}](D\dot{\boldsymbol{r}} + \boldsymbol{\nabla}_{\boldsymbol{r}} \times \boldsymbol{m}_{\boldsymbol{k}})f, \tag{S8}$$

with $[d\boldsymbol{k}] = d\boldsymbol{k}/(2\pi)^3$ and f the field-induced distribution function. The second term on the right-hand side only contributes to generate magnetization currents. As our experiment only measures transport currents, we drop it from now on. The factor

$$D = 1 + \frac{e}{\hbar}\boldsymbol{B} \cdot \boldsymbol{\Omega}_{\boldsymbol{k}}, \tag{S9}$$

takes into account the change of the volume of the phase space in an applied magnetic field [18]. It is convenient to rewrite the equations of motion (S4) as

$$\dot{\boldsymbol{r}} = \frac{1}{\hbar D}[\boldsymbol{\nabla}_{\boldsymbol{k}}\epsilon_{\boldsymbol{k}} + e\boldsymbol{E}\times\boldsymbol{\Omega}_{\boldsymbol{k}} + \frac{e}{\hbar}(\boldsymbol{\nabla}_{\boldsymbol{k}}\epsilon_{\boldsymbol{k}}\cdot\boldsymbol{\Omega}_{\boldsymbol{k}})\boldsymbol{B}], \tag{S10a}$$

$$\hbar\dot{\boldsymbol{k}} = \frac{1}{D}[-e\boldsymbol{E} - \frac{e}{\hbar}\boldsymbol{\nabla}_{\boldsymbol{k}}\epsilon_{\boldsymbol{k}}\times\boldsymbol{B} - \frac{e^2}{\hbar}(\boldsymbol{E}\cdot\boldsymbol{B})\boldsymbol{\Omega}_{\boldsymbol{k}}] \tag{S10b}$$

Using first equation above we can write (S8) as

$$\boldsymbol{j} = -e\int[d\boldsymbol{k}][\tilde{\boldsymbol{v}}_{\boldsymbol{k}} + \frac{e}{\hbar}\boldsymbol{E}\times\boldsymbol{\Omega}_{\boldsymbol{k}} + \frac{e}{\hbar}(\tilde{\boldsymbol{v}}_{\boldsymbol{k}}\cdot\boldsymbol{\Omega}_{\boldsymbol{k}})\boldsymbol{B}]f, \tag{S11}$$

where we defined the generalized velocity

$$\tilde{\boldsymbol{v}}_{\boldsymbol{k}} = \boldsymbol{v}_{\boldsymbol{k}} - (1/\hbar)\boldsymbol{\nabla}_{\boldsymbol{k}}(\boldsymbol{m}\cdot\boldsymbol{B}), \tag{S12}$$

in terms of the zero-field velocity $\boldsymbol{v}_{\boldsymbol{k}} = (1/\hbar)\boldsymbol{\nabla}_{\boldsymbol{k}}\epsilon^0_{\boldsymbol{k}}$.

The Boltzmann equation determines the Fermi distribution function in terms of the fields. In the steady state, and for a uniform system we may write it as

$$\dot{\boldsymbol{k}}\cdot\boldsymbol{\nabla}_{\boldsymbol{k}}f = I_{\text{coll}}. \tag{S13}$$

For the right-hand side we assume the relaxation time approximation, where the collision integral is [16]

$$I_{\text{coll}} = -\frac{f-f_0}{\tau} + \left(1-\frac{\tau}{\tau_v}\right)\frac{\delta\mu}{\tau}\partial_\epsilon f_0. \tag{S14}$$

Here, τ_v is the internode scattering and τ is the intranode scattering, while $\delta\mu$ accounts for the potential chemical potential shift between the two valleys, as in Ref. [16]. We will assume that $\delta\mu = c_a\mathbf{E}\cdot\mathbf{B}$, where c_a is a chiral anomaly coefficient proportional to the internode scattering time τ_v [16].

We are interested in finding a solution of the form $f = f_0 + f_1$ such that

$$f_0(\epsilon_{\boldsymbol{k}} - E_F) = \theta(E_F - \epsilon^0_{\boldsymbol{k}} - \boldsymbol{m}_{\boldsymbol{k}}\cdot\boldsymbol{B}) \tag{S15}$$

is the equilibrium distribution function for a Fermi energy E_F at $T=0$, defined by the step-function $\theta(x) = 0(x<0), 1(x\geq 0)$. We emphasize that $\epsilon_{\boldsymbol{k}}$ depends on magnetic field already through (S7).

Solving (S13) using (S10b) we obtain

$$\begin{aligned} -\frac{e}{D}[\boldsymbol{E} + \frac{e}{\hbar}(\boldsymbol{E}\cdot\boldsymbol{B})\boldsymbol{\Omega}_{\boldsymbol{k}}]\cdot\boldsymbol{\nabla}_{\boldsymbol{p}}f_0 - \frac{e}{D}\left(\boldsymbol{v}_{\boldsymbol{k}}\times\boldsymbol{B}\right)\cdot\boldsymbol{\nabla}_{\boldsymbol{p}}f_1 \\ = -\frac{f_1}{\tau} + \left(1-\frac{\tau}{\tau_v}\right)\frac{\delta\mu}{\tau}\partial_\epsilon f_0, \end{aligned} \tag{S16}$$

where we defined for convenience $\boldsymbol{\nabla_p} = (1/\hbar)\boldsymbol{\nabla_k}$. We note that $\boldsymbol{\nabla_p} f_0 = (1/\hbar)(\boldsymbol{\nabla_k}\epsilon_{\boldsymbol{k}})\partial_\epsilon f_0$. We can solve Eq. (S16) recursively as [16]

$$\begin{aligned} f_1 = &\sum_{i=0} \left(\frac{\tau e}{D}\right)^i \hat{L}^i \left[\left(\frac{\tau e}{D}\right)\left(\boldsymbol{E} + \frac{e}{\hbar}(\boldsymbol{E}\cdot\boldsymbol{B})\boldsymbol{\Omega_k}\right)\right] \cdot \boldsymbol{\nabla_p} f_0 \\ &- \left(1 - \frac{\tau}{\tau_v}\right)\delta\mu\partial_\epsilon f_0, \end{aligned} \tag{S17}$$

where $\hat{L} = (\boldsymbol{v_k} \times \boldsymbol{B}) \cdot \boldsymbol{\nabla_p}$ is the Lorentz operator. We are now in position to write the current to linear order in the electric field. We separate the current terms in powers of the scattering time up to second order

$$\boldsymbol{j} = \boldsymbol{j}_0 + \boldsymbol{j}_1 + \boldsymbol{j}_2 \tag{S18}$$

$$\boldsymbol{j}_0 = -e\int_{\mathrm{BZ}}[d\boldsymbol{k}]\frac{e}{\hbar}\boldsymbol{E}\times\boldsymbol{\Omega_k} f_0 \tag{S19}$$

$$\boldsymbol{j_1} = -e\tau\int_{\mathrm{BZ}}[d\boldsymbol{k}]\left\{\frac{1}{D}\left[\tilde{\boldsymbol{v}}_{\boldsymbol{k}} + \frac{e}{\hbar}(\boldsymbol{\Omega_k}\cdot\tilde{\boldsymbol{v}}_{\boldsymbol{k}})\boldsymbol{B}\right]\left[(e\tilde{\boldsymbol{v}}_{\boldsymbol{k}} + \frac{\tau_c}{\tau}\frac{e^2}{\hbar}(\boldsymbol{\Omega_k}\cdot\tilde{\boldsymbol{v}}_{\boldsymbol{k}})\boldsymbol{B})\cdot\boldsymbol{E}\partial_\epsilon f_0\right]\right\}, \tag{S20}$$

$$\boldsymbol{j_2} = -e\tau^2\int_{\mathrm{BZ}}[d\boldsymbol{k}]\left\{\frac{1}{D}\left[\tilde{\boldsymbol{v}}_{\boldsymbol{k}} + \frac{e}{\hbar}(\boldsymbol{\Omega_k}\cdot\tilde{\boldsymbol{v}}_{\boldsymbol{k}})\boldsymbol{B}\right]\left[\frac{1}{D}\hat{L}(e\tilde{\boldsymbol{v}}_{\boldsymbol{k}} + \frac{e^2}{\hbar}(\boldsymbol{\Omega_k}\cdot\tilde{\boldsymbol{v}}_{\boldsymbol{k}})\boldsymbol{B})\cdot\boldsymbol{E}\partial_\epsilon f_0\right]\right\} \tag{S21}$$

The term $\boldsymbol{j}_0$ encodes a Hall effect. Because Eq. (S15) depends on magnetic field and the orbital moment, the Hall effect in $\boldsymbol{j}_0$ has different contributions. At zero magnetic field, the dependence on the orbital magnetization drops and $\boldsymbol{j}_1$ is only non-zero if the material breaks-time reversal symmetry. Such anomalous Hall effect vanishes for time-reversal symmetric systems such as CoSi. At finite magnetic field, $\boldsymbol{j}_0$ vanishes only in the absence of orbital magnetization, even when the material respects time-reversal symmetry. When inversion symmetry is broken, a nonzero orbital magnetization generates an intrinsic Hall current proportional to $\boldsymbol{B}$, derived in Ref. [19] and later calculated for a single Weyl node in Ref. [20]. This contribution, which we call orbital-magnetic-moment Hall effect, arises from a k-asymmetry in the energy spectrum due to the magnetic field, as given by (S7) [21].

The term $\boldsymbol{j}_1$ contains terms proportional to a scattering time. These will result in a longitudinal magnetoresistance, and will include Drude contributions, Berry curvature contributions, and orbital magnetic moment contributions. To obtain the last term in $\boldsymbol{j}_1$, we noticed that $\delta\mu$ can be found self-consistently to be proportional to $\mathbf{E}\cdot\mathbf{B}$, since $\delta\mu = c\tau_v\mathbf{E}\cdot\mathbf{B}$, where c is a constant [14, 16]. Hence, the terms contributing to $\boldsymbol{j}_1$ coming from the second line in Eq. (S17) and the last term in the first line in Eq. (S17) can be combined into a single

term by redefining a new scattering time τ_c. We call τ_c the chiral scattering time, which in general depends on both the inter and intranode scattering. When $\tau \ll \tau_v$, $\tau_c \approx \tau_v$. This assumption is well justified since the multi-fold fermions are maximally separated in momentum space, and we will adopt it in what follows. We determine the ratio τ_v/τ experimentally, as discussed in the main text and below.

The term $\boldsymbol{j}_2$, proportional to τ^2, includes the classical Hall effect due the Lorentz force. We now calculate all contributions for the multifold fermions of CoSi.

A. Angle-dependent magnetoconductance of CoSi

At the R point, and neglecting spin-orbit coupling, as reasonable for CoSi [22, 23], there are two Weyl points of the same chirality, doped above the crossing point ($\mu > 0$). The Hamiltonian to linear order in momentum for such a *double Weyl* fermion reads

$$H^{(dW)} = \chi_R v_R \boldsymbol{\sigma} \cdot \boldsymbol{k} \otimes \tau_0 \otimes s_0, \tag{S22}$$

where v_R is the Fermi velocity at the R point, $\chi_R = \pm 1$ is the chirality of the R node and σ is a vector of Pauli matrices representing an orbital-degree of freedom. Using τ_0 we encode the valley degeneracy, i.e. the R point is composed of two Weyls of the same chirality, and s_0 we encode the spin degeneracy. For a single Weyl fermion, the Berry curvature and orbital magnetic moment of each of the bands are

$$\boldsymbol{\Omega}_{\boldsymbol{k}} = -\chi_R \frac{s}{2k^2} \hat{\boldsymbol{k}}, \tag{S23a}$$

$$\boldsymbol{m}_{\boldsymbol{k}} = -\chi_R \frac{e v_R}{2k} \hat{\boldsymbol{k}}, \tag{S23b}$$

where $\hat{\boldsymbol{k}}$ is the unit vector along $\boldsymbol{k}$, and $s = -1, +1$ for the valence and conduction band, respectively. While the Berry curvature changes sign for the each band, the orbital magnetic moment does not.

Consistent with all experimental reports of CoSi samples [23–25] and our quantum oscillations data we assume that R point is filled above the node up to its conduction band, with Fermi momentum k_F^R, and we assign it a chirality $\chi_R = +1$.

We assume an electric field along the x direction, $\boldsymbol{E} = (E_x, 0, 0)$, and vary the magnetic field by an angle θ with respect to the x direction, $\boldsymbol{B} = B_0(\cos\theta, 0, \sin\theta)$. The longitudinal conductivity for the component of the current along the electric field for the double Weyl

at R is entirely due to Eq. (S20). The corresponding current density $j_x^{(dW)}$ and conductivity $\sigma_{xx}^{(dW)}$ read

$$j_x^{(dW)} = \sigma_{xx}^{(dW)} E_x \tag{S24a}$$

$$\sigma_{xx}^{(dW)} = 4\left[\frac{\tau e^2 v_R (k_F^R)^2}{6\pi^2\hbar} + \frac{\left[(1+\frac{5\tau_v}{\tau})\cos^2\theta - 2\right]\tau e^4 v_R B_0^2}{120\pi^2\hbar^3 (k_F^R)^2}\right] \tag{S24b}$$

The factor 4 in front of the squared brackets takes into account the existence of two Weyl nodes at R and the spin degeneracy. As we sweep the angle from $\theta = 0$ to $\theta = \pi/2$ the longitudinal magnetoconductance goes from being positive to negative due to the second term on the right-hand side. The chiral anomaly contributes to this term via the last term in Eq. (S20), via the term proportional to τ_v/τ. It has a $\cos^2\theta$ angular dependence as observed in our experiment. Later we will fix τ_v/τ with our experiments, which suggest $\tau_v/\tau \gg 1$. However, if we assume $\tau = \tau_v$ Eq.(S24b) recovers the results of Ref. [12], consistent with the fact that this reference did not distinguish inter and intranode scattering.

To linear order in momentum, the *threefold fermion* at Γ is described by the Hamiltonian

$$H^{(3f)} = \chi_\Gamma v_\Gamma \boldsymbol{S}\cdot\boldsymbol{k}\otimes s_0, \tag{S25}$$

where v_Γ is the Fermi velocity, $\boldsymbol{S}$ is a vector of spin-1 matrices representing the orbital-degree of freedom, and $\chi_\Gamma = \pm 1$ is the chirality of the Γ node. Using s_0 we encode again the spin degeneracy. Eq. (S25) needs to describe a threefold Fermion with the opposite chirality compared to (S22), as enforced by the Nielsen-Ninomiya, or fermion doubling, theorem [4, 5], *i.e.* $\chi_\Gamma = -\chi_R$.

The threefold Hamiltonian has three bands which are spin-degenerate. The corresponding Berry curvature and orbital magnetic were calculated in Ref. [2] and are

$$\boldsymbol{\Omega}_{\boldsymbol{k}}^n = \eta_\Omega^n \chi_\Gamma \frac{1}{2k^2}\hat{\boldsymbol{k}}, \tag{S26a}$$

$$\boldsymbol{m}_{\boldsymbol{k}}^n = \eta_m^n \chi_\Gamma \frac{e v_\Gamma}{2k}\hat{\boldsymbol{k}}, \tag{S26b}$$

where n labels the bottom, middle and upper band of the threefold fermion, $n = 1, 2, 3$ respectively. Unlike the double-Weyl node, where the Chern numbers of the band are $2\times(1,-1)$, the threefold bands have Chern numbers $2, 0, -2$. Hence $\eta_\Omega^n = (-2, 0, 2)$ for the top, middle and bottom bands, respectively. The orbital magnetic moment of top and bottom

bands equals that of a Weyl fermion. For the middle band, the orbital magnetic moment is twice that of a Weyl fermion. This results in $\eta_m^n = (1, 2, 1)$ [2].

Next, we assume that the chemical potential crosses the lower two bands, the linearly dispersing band ($n = 1$) and the central quadratic band ($n = 2$), *i.e.* we assume $\mu_{3f} < 0$ measured with respect to the threefold crossing. We label the corresponding Fermi momentum of the linear band k_F^Γ. Since the middle band is flat in the linear approximation, we promote it to a quadratic, hole-like band, for it to cross the Fermi level. We call its Fermi momentum $k_F^q \gg k_F^\Gamma$, see Fig. 4. These assumptions are justified by previously reported density functional theory (DFT) calculations and ARPES experiments [23–25].

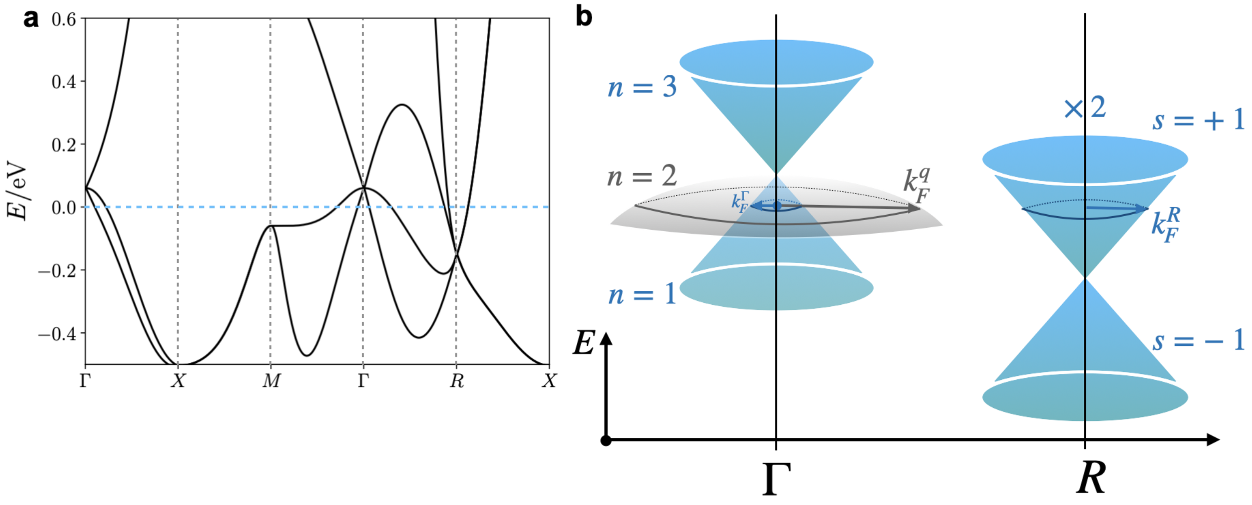

Supplementary Figure 4: **Low energy semiclassical theory. a** Tight-binding band structure energy versus momentum of CoSi crystal. **b** Schematic of energy versus momentum of the threefold fermion located at Γ and the fourfold fermion located at R (two Weyl cones of equal chirality). In our semiclassical calculation we assume that $k_F^q, k_F^R \gg k_F^\Gamma$, consistent with previous experiments [23–25] and our quantum-oscillation data. Ignoring spin, $n = 1, 2, 3$ labels the three bands at Γ, while $s = -1, 1$ labels the valence and conduction bands at R, respectively, each doubly degenerate (indicated with a ×2 in the figure). Their Berry curvatures and orbital magnetic moments are given by Eqs. (S23) and (S26). Considering spin we add an additional two-fold degeneracy for all bands at Γ and R.

We first calculate the magneto-conductivity of the $n = 1$ setting the chirality in (S25) to be the opposite of that of R, i.e. $\chi_\Gamma = -1$. Assuming the same configuration of electric

and magnetic fields as above and using Eq. (S20) the component of the current along the electric field for the threefold fermion at Γ is

$$j_x^{(3f)} = \sigma_{xx}^{(3f)} E_x, \tag{S27a}$$

$$\sigma_{xx}^{(3f)} = 2\left[\frac{\tau e^2 v_\Gamma (k_F^\Gamma)^2}{6\pi^2\hbar} + \frac{\left[(-9+30\frac{\tau_v}{\tau})\cos^2\theta - 2\right]\tau e^4 v_\Gamma B_0^2}{120\pi^2\hbar^3 (k_F^\Gamma)^2}\right]. \tag{S27b}$$

The factor 2 in front of this expression takes into account the spin degeneracy. As a benchmark, we note that setting the magnetic moment to zero and $\theta = 0$ we recover the expression given by [11]. However, as we emphasized in the main text, since the magnetic moment is as sizable as the Berry curvature, it cannot be neglected.

Lastly, using the same formalism we can calculate the contribution of the central parabolic band. As it is a quadratic band with no Chern number, it is expected to lead to an ordinary negative magnetoconductance (positive magnetoresistance). Its magnitude is inversely proportional to the Fermi momentum k_F^q of the quadratic band, i.e. $\sigma \propto \frac{e^4\tau(v_\Gamma)^2 B_0^2}{\hbar^2 E_F k_F^q}$, where v_Γ is the Fermi velocity of the linear bands. Compared to the linearly dispersing bands, the quadratic band has a relatively large E_F and k_F^q, which allows us to neglect its positive contribution to magneto-conductivity.

Defining the magneto-conductance as

$$\sigma_{PMC}(B_0) \equiv \frac{\sigma(B_0) - \sigma(B_0 = 0)}{\sigma(B_0 = 0)} \tag{S28}$$

Combining the contribution from both multifold fermions we have

$$\sigma_{PMC}(B_0) = \frac{\left[\left(-9 + 2r_1 + 10\,(3 + r_1)\,\frac{\tau_v}{\tau}\right)\cos^2\theta - (2 + 4r_1)\right]}{20\,(1 + 2r_2)}\,\frac{B_0^2}{(k_F^\Gamma)^4}\,\frac{e^2}{\hbar^2}. \tag{S29}$$

with $r_1 = \left(\frac{k_F^\Gamma}{k_F^R}\right)^2 \frac{v_R}{v_\Gamma}$ and $r_2 = \left(\frac{k_F^R}{k_F^\Gamma}\right)^2 \frac{v_R}{v_\Gamma}$, resulting in Eq.(1) in the main text. To estimate the positive magnetoconductance we can use that tight-binding models fit well the DFT dispersion if $v_R = v_\Gamma/\sqrt{3}$ and that from quantum oscillations $k_F^R \approx 5k_F^\Gamma$. In the longitudinal configuration ($\theta = 0$) this simplifies to

$$\sigma_{PMC}(B_0)\Big|_{\theta=0} \approx \frac{\left[-825 - 2\sqrt{3} + 10\left(225 + \sqrt{3}\right)\frac{\tau_v}{\tau}\right] e^2 B_0^2}{500\left(3 + 50\sqrt{3}\right)\hbar^2\left(k_F^\Gamma\right)^4}. \tag{S30}$$

We can use $k_F^\Gamma = 0.25\text{nm}^{-1}$ to get

$$\sigma_{PMC}(B_0 = 1T, \theta = 0) \approx -0.001\% + 0.003\% \frac{\tau_v}{\tau}. \tag{S31}$$

We note that when the internode scattering time is slower than the intranode scattering time, $\frac{\tau_v}{\tau} \ll 1$, the magnetoconductance becomes negative. This is consistent with the physical intuition that the anomalous positive magnetoresistance occurs only when internode scattering dominates, i.e. $\frac{\tau_v}{\tau} \gg 1$. By treating $\frac{\tau_v}{\tau}$ as a fitting parameter, we can include other trivial scattering terms that we have neglected in the calculation, e.g. between Weyl and trivial bands that lead to positive magnetoresistance [26].

With the above we need that $\frac{\tau_v}{\tau} \approx 10^2$ to get an enhancement of 1% at 1T as seen experiment. That $\frac{\tau_v}{\tau} \gg 1$ is reasonable, since the chiral multifold fermions are maximally separated in the Brillouin zone.

We have checked that for both the Γ and the R points the orbital magnetic moment terms always contribute to decrease the magneto-conductance, *i.e.* they result in positive magnetoresistance. Therefore, their effect is to reduce the chiral anomaly negative magnetoresistance, but their magnitude is insufficient to overcome it.

VI. NON-LINEAR CONTRIBUTION TO THE HALL EFFECT

This section contains the main theoretical results behind our interpretation of the Hall conductivity. The large orbital magnetic moment and Berry curvature close to the node at Γ are the main contributors to a cubic-in-magnetic field contribution to the Hall effect. This result is a consequence of the large orbital magnetic moment and Berry curvature close to the Γ point.

We wish to argue why we can expect the cubic terms of the Hall effect to follow the positive magneto-conductivity, i.e. magnetic fields where we observe positive magnetoconductance have also an observable cubic contribution to the Hall effect.

First let us discuss why we expect non-linear Hall terms in the semiclassical picture. The Hall response is determined solely by the intrinsic (i.e. τ independent) Eq. (S19) and the Lorentz part Eq. (S21), proportional to τ^2. The dominant linear-in-B term, that we subtract in the main text to highlight the cubic contribution, is due to the Lorentz force. It is proportional to the density of carriers, and it entirely determined by Eq. (S21). After the linear-in-B contribution is subtracted, both Eq. (S19) and Eq. (S21) contribute to the

cubic B^3-dependent part of the Hall conductivity.

In the following we will again assume $\boldsymbol{E} = (E_x, 0, 0)$, and vary the magnetic field by an angle θ with respect to the x direction, $\boldsymbol{B} = B_0(\cos\theta, 0, \sin\theta)$. Contrary to the previous section, we will here be interested in the y component of the current, j_y, which is perpendicular to $\boldsymbol{E}$. We now analyze the separate contributions and determine the dominant cubic terms.

A. Threefold at Γ

The three-fold fermion at the Γ point has three bands, a central quadratic band that is relatively flat (has a large effective mass), and two linearly dispersing bands. The central quadratic band has zero Berry curvature and finite orbital angular momentum while the linearly dispersing bands have both Berry curvature and finite orbital angular momentum, as per Eq. (S26). Consistent with our quantum oscillation data, previous photoemission [24] and optical experiments [23, 25] we assume that the lowest ($n = 1$) and central ($n = 2$) Γ bands are partially occupied, with momentum k_F^Γ and k_F^q respectively (see Fig. 4).

The bottom linear band contributes through Eq. (S21)

$$j_{2,y} = -\frac{(e^3 \sin\theta(e^2 B_0^2 + 20\hbar^2 (k_F^\Gamma)^4)\tau^2 v_\Gamma^2) B_0 E_x}{60\pi^2\hbar^4 (k_F^\Gamma)^3}, \tag{S32}$$

and through Eq. (S19)

$$j_{0,y} = -\frac{e^3(9B_0^2 e^2 + 5\hbar^2 (k_F^\Gamma)^4) B_0 E_x \sin\theta}{30\pi^2\hbar^4 (k_F^\Gamma)^5}, \tag{S33}$$

where we have taken into account the spin degeneracy.

The central quadratic band at Γ has zero orbital magnetic moment and which, combined with time-reversal symmetry, renders $j_{0,y} = 0$, from Eq. (S19). Through Eq. (S21) this band contributes

$$j_{2,y} = -\frac{e^3 v_\Gamma^2 \tau^2 \left(\frac{4(E_F^q k_F^q)^2}{v_\Gamma^2} + B_0^2 e^2\right) B_0 E_x \sin\theta}{3\pi^2\hbar^4 (k_F^q)^3}. \tag{S34}$$

where, as we did previously, we assumed a hole-like parabolic dispersion with Fermi momentum k_F^q and Fermi energy E_F^q, and have taken into account the spin degeneracy.

B. Fourfold at R

The two top linear bands contribute through Eq. (S21) as

$$j_{2,y} = \frac{-4\tau^2 e^3 v_R^2 \left(e^2 B_0^2 - 5\hbar^2 (k_F^R)^4\right) B_0 E_x \sin\theta}{30\pi^2 \hbar^4 (k_F^R)^3}, \tag{S35}$$

and the contribution from Eq. (S19) is

$$j_{0,y} = \frac{-4e^3 \left(9e^2 B_0^2 + 5\hbar^2 (k_F^R)^4\right) B_0 E_x \sin\theta}{120\pi^2 \hbar^4 (k_F^R)^5}. \tag{S36}$$

As with the longitudinal conductivity, the factor 4 takes into account the double Weyl node structure and the spin degeneracy.

C. Dominant contribution to the cubic terms in the Hall conductivity

In the main text we subtract the linear Hall conductivity and are left with a cubic contribution $\sigma_{xy} \propto B_0^3$. Experimentally, the central quadratic band at Γ and the R-point double Weyl form the largest Fermi-surfaces (see discussion in main text, Fig. 1a, and e.g.[24, 27]). Hence, we have $k_F^q, k_F^R \gg k_F^\Gamma$. With this condition, assuming the same order of magnitude for τ in all bands, the main contribution to the cubic term in σ_{xy} comes from Eqs. (S32) and (S33). Both of these equations are finite due to the orbital magnetic moment and the Berry curvature of the linear band around Γ. Since these quantities diverge close to the multifold crossing point at Γ, see Eq. (S26), we conclude that the dominant contribution to the cubic term in the non-linear Hall is the filled linear band at Γ. The positive magnetoconductance is also determined by the Berry curvature and orbital magnetic moment close to the node. Hence, it is reasonable that the cubic Hall terms and positive magneto-conductance are sizable around the same values of magnetic field, as observed in our experimental data.

VII. SUPPLEMENTARY REFERENCES

[1] B. Bradlyn, J. Cano, Z. Wang, M. G. Vergniory, C. Felser, R. J. Cava, and B. A. Bernevig, Beyond dirac and weyl fermions: Unconventional quasiparticles in conventional crystals, Science

353, aaf5037 (2016), https://www.science.org/doi/pdf/10.1126/science.aaf5037.

[2] F. Flicker, F. de Juan, B. Bradlyn, T. Morimoto, M. G. Vergniory, and A. G. Grushin, Chiral optical response of multifold fermions, Phys. Rev. B **98**, 155145 (2018).

[3] M. Ezawa, Chiral anomaly enhancement and photoirradiation effects in multiband touching fermion systems, Phys. Rev. B **95**, 205201 (2017).

[4] H. B. Nielsen and M. Ninomiya, Absence of neutrinos on a lattice: (I). Proof by homotopy theory, Nuclear Physics B **185**, 20 (1981).

[5] H. B. Nielsen and M. Ninomiya, Absence of neutrinos on a lattice: (II). Intuitive topological proof, Nuclear Physics B **193**, 173 (1981).

[6] L. Lepori, M. Burrello, and E. Guadagnini, Axial anomaly in multi-weyl and triple-point semimetals, Journal of High Energy Physics **2018**, 110 (2018).

[7] D. T. Son and B. Z. Spivak, Chiral anomaly and classical negative magnetoresistance of Weyl metals, Phys. Rev. B **88**, 104412 (2013), publisher: American Physical Society.

[8] R. Lundgren, P. Laurell, and G. A. Fiete, Thermoelectric properties of weyl and dirac semimetals, Phys. Rev. B **90**, 165115 (2014).

[9] K.-S. Kim, H.-J. Kim, and M. Sasaki, Boltzmann equation approach to anomalous transport in a weyl metal, Phys. Rev. B **89**, 195137 (2014).

[10] J. Ma and D. A. Pesin, Chiral magnetic effect and natural optical activity in metals with or without weyl points, Phys. Rev. B **92**, 235205 (2015).

[11] S. Nandy, S. Manna, D. Călugăru, and B. Roy, Generalized triple-component fermions: Lattice model, fermi arcs, and anomalous transport, Phys. Rev. B **100**, 235201 (2019).

[12] T. Morimoto, S. Zhong, J. Orenstein, and J. E. Moore, Semiclassical theory of nonlinear magneto-optical responses with applications to topological dirac/weyl semimetals, Phys. Rev. B **94**, 245121 (2016).

[13] M. Imran and S. Hershfield, Berry curvature force and lorentz force comparison in the magnetotransport of weyl semimetals, Phys. Rev. B **98**, 205139 (2018).

[14] M.-X. Deng, H.-J. Duan, W. Luo, W. Y. Deng, R.-Q. Wang, and L. Sheng, Quantum oscillation modulated angular dependence of the positive longitudinal magnetoconductivity and planar hall effect in weyl semimetals, Phys. Rev. B **99**, 165146 (2019).

[15] M.-X. Deng, J.-Y. Ba, R. Ma, W. Luo, R.-Q. Wang, L. Sheng, and D. Y. Xing, Chiral-anomaly-induced angular narrowing of the positive longitudinal magnetoconductivity in weyl

semimetals, Phys. Rev. Res. **2**, 033346 (2020).

[16] D. Mandal, K. Das, and A. Agarwal, Chiral anomaly and nonlinear magnetotransport in time reversal symmetric Weyl semimetals, Physical Review B **106**, 035423 (2022), 2201.02505.

[17] G. Sundaram and Q. Niu, Wave-packet dynamics in slowly perturbed crystals: Gradient corrections and Berry-phase effects, Physical Review B **59**, 14915 (1999).

[18] D. Xiao, M.-C. Chang, and Q. Niu, Berry phase effects on electronic properties, Reviews of Modern Physics **82**, 1959 (2010).

[19] Y. Gao, S. A. Yang, and Q. Niu, Field induced positional shift of bloch electrons and its dynamical implications, Phys. Rev. Lett. **112**, 166601 (2014).

[20] K. Das and A. Agarwal, Intrinsic hall conductivities induced by the orbital magnetic moment, Phys. Rev. B **103**, 125432 (2021).

[21] T. Cai, S. A. Yang, X. Li, F. Zhang, J. Shi, W. Yao, and Q. Niu, Magnetic control of the valley degree of freedom of massive dirac fermions with application to transition metal dichalcogenides, Phys. Rev. B **88**, 115140 (2013).

[22] G. Chang, S.-Y. Xu, B. J. Wieder, D. S. Sanchez, S.-M. Huang, I. Belopolski, T.-R. Chang, S. Zhang, A. Bansil, H. Lin, and M. Z. Hasan, Unconventional Chiral Fermions and Large Topological Fermi Arcs in RhSi, Phys. Rev. Lett. **119**, 206401 (2017), publisher: American Physical Society.

[23] Z. Ni, K. Wang, Y. Zhang, O. Pozo, B. Xu, X. Han, K. Manna, J. Paglione, C. Felser, A. G. Grushin, F. d. Juan, E. J. Mele, and L. Wu, Giant topological longitudinal circular photo-galvanic effect in the chiral multifold semimetal CoSi, Nature Communications **12**, R935 (2021).

[24] D. Takane, Z. Wang, S. Souma, K. Nakayama, T. Nakamura, H. Oinuma, Y. Nakata, H. Iwasawa, C. Cacho, T. Kim, K. Horiba, H. Kumigashira, T. Takahashi, Y. Ando, and T. Sato, Observation of Chiral Fermions with a Large Topological Charge and and Associated Fermi-Arc Surface States in CoSi, Phys. Rev. Lett. **122**, 076402 (2019), arXiv:1809.01312 [cond-mat].

[25] B. Xu, Z. Fang, M.-A. Sanchez-Martinez, J. W. F. Venderbos, Z. Ni, T. Qiu, K. Manna, K. Wang, J. Paglione, C. Bernhard, C. Felser, E. J. Mele, A. G. Grushin, A. M. Rappe, and L. Wu, Optical signatures of multifold fermions in the chiral topological semimetal CoSi, Proceedings of the National Academy of Sciences **83**, 202010752 (2020).

[26] J. Suh and H. Min, Effect of trivial bands on chiral anomaly-induced longitudinal magnetoconductivity in weyl semimetals, arXiv:2401.13855 (2024), arXiv:2401.13855 [cond-mat.mes-hall].

[27] N. Huber, V. Leeb, A. Bauer, G. Benka, J. Knolle, C. Pfleiderer, and M. A. Wilde, Quantum oscillations of the quasiparticle lifetime in a metal, Nature **621**, 276 (2023), 2306.09420.
